# Perceived mental strain dissociates from perceived physical strain during relative intensity submaximal exercise on ascent from low to high altitude

**DOI:** 10.14814/phy2.14934

**Published:** 2021-07-07

**Authors:** Patrick J. Drouin, Jeremy J. Walsh, Jeroen Swart, Trevor A. Day, Michael E. Tschakovsky

**Affiliations:** ^1^ Human Vascular Control Laboratory School of Kinesiology and Health Studies Queen's University Kingston ON Canada; ^2^ Exercise Metabolism and Inflammation Laboratory University of British Columbia Okanagan Kelowna BC Canada; ^3^ Department of Kinesiology McMaster University Hamilton ON Canada; ^4^ UCT/MRC Research Unit for Exercise Science and Sports Medicine Department of Human Biology Sports Science Institute of South Africa University of Cape Town Cape Town South Africa; ^5^ Department of Biology Faculty of Science and Technology Mount Royal University Calgary AB Canada

**Keywords:** altitude, perceived mental strain, perceived physical strain, submaximal exercise

## Abstract

Perceived fatigability, which has perception of physical strain and of mental strain as its components, can impact exercise tolerance. Upon ascent to high altitude, low landers experience reduced exercise capacity and reduced tolerance for a given absolute submaximal work rate. It is established that perceived physical strain tracks with relative exercise intensity. However, it is not known how altitude ascent affects perceived mental strain relative to perceived physical strain. We tested the hypothesis that when exercising at the same relative exercise intensity perceived physical strain will remain unchanged whereas perceived mental strain will decrease on ascent from low to high altitude in the Everest region in Nepal. Twelve hours after arriving at each of three elevations; 1400 m, 3440 m, and 4240 m, 12 untrained participants used the task effort awareness (TEA) and physical‐rating of perceived exertion (P‐RPE) scales to report perceived mental and physical strain during a 20 min walking test at a self‐monitored heart rate reserve (HRR) range of 40–60% (Polar HR Monitor). TEA and P‐RPE were recorded twice during exercise (5–7 min and 14–16 min). Neither P‐RPE (1400 m: 11.1 ± 1.8, 3440 m: 10.7 ± 1.2, 4240 m: 11.5 ± 1.5) nor %HRR (1400 m: 55.25 ± 7.34, 3440 m: 51.70 ± 6.70, 4240 m: 50.17 ± 4.02) changed as altitude increased. TEA decreased at 4240 m (2.05 ± 0.71) compared to 1400 m (3.44 ± 0.84)––this change was not correlated with any change in %HRR nor was it due to a change in core affect. These findings support our hypothesis and demonstrate the independence of perceived physical and perceived mental strain components of perceived fatigability. Implications for exercise tolerance remain to be determined.

## Graphical abstract

Until now, no work had examined the domain of perceived mental strain upon ascent from low to high altitude. We tested and confirmed the hypothesis that at the same relative submaximal exercise intensity, participants would perceive the same amount of physical strain but less mental strain upon ascent from low to high altitude. Therefore, perceived mental strain dissociates from perceived physical strain upon ascent from low to high altitude—the mechanism(s) responsible for this dissociation remain to be determined.

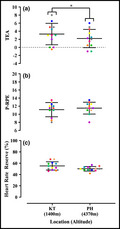

## 1 INTRODUCTION

Ascent to high altitude—whether for work, leisure, or sport—is becoming more accessible and more common. Upon ascent to high altitude, low landers experience reduced exercise capacity and reduced tolerance for a given absolute submaximal work rate (Aliverti et al., 2011). We know that central neural and peripheral excitation–contraction coupling mechanisms of fatigue are exacerbated with the hypoxia accompanying high altitude and contribute to reduced exercise tolerance (Grocott et al., 2019). For example, when exposed to acute and chronic high altitude hypoxia, peripheral, and central fatigue were both exacerbated following a constant load submaximal cycling test (Amann et al., 2013). Importantly, fatigue is not only about the physiological mechanisms of compromised central neural and peripheral skeletal muscle excitation‐contraction coupling, but also includes aspects of perception (Kluger et al., 2013). Specifically, Kluger et al. (2013) proposed a taxonomy for characterizing fatigue in which performance fatigability characterizes the central and peripheral physiological mechanisms contributing to fatigue whereas perceived fatigability refers to the “subjective sensations of weariness, increasing sense of effort, mismatch between effort expended and actual performance, or exhaustion.” Building upon the work of Kluger et al. (2013), Venhorst et al. (2018b), developed a three‐dimensional framework for the domain of perceived fatigability. Under the sensory‐discriminatory dimension, Venhorst et al. (2018b) propose that perceived strain (formerly referred to as perceived exertion) be broken down into distinct categories: (1) perceived physical strain, referring to the “subjective overall perception of physical strain from central and peripheral sites” and (2) perceived mental strain (commonly referred to as perceived effort or mental effort) referring to the “subjective awareness of mental effort required to perform the task at hand.”

It is thought that perceived mental strain is determined by a corollary of the central motor drive sent from the brain to the muscle determining muscle recruitment (Christian et al., 2014; Hureau et al., 2018; Marcora, 2009; Proske, 2005). de Morree et al. (2012) most clearly demonstrated this phenomenon by simultaneously measuring movement‐related cortical potentials and perceived mental strain during bicep exercise. Participants reported their perception of mental strain using a modified Borg category ratio 10 scale and ensured that participants’ rating of perceived mental strain was “based exclusively on effort and not on any burning sensation in the arms”––clearly distinguishing perceived mental strain from perceived physical strain (de Morree et al., 2012). de Morree et al. (2012) demonstrated that if central motor drive increases, so does perceived mental strain. As opposed to perceived mental strain, perceived physical strain reflects sensations and feelings of physical stress and fatigue during exercise, which are sensed primarily via afferent (group III and IV) feedback from active and inactive tissue during exercise (Hureau et al., 2018; Venhorst et al., 2018a, 2018b).

In contrast to performance fatigability at high altitude, we know very little about perceived fatigability. Investigations into perceived physical strain at high altitude have found that for the same perceived physical strain the absolute work rate performed is always reduced––as is peak exercise capacity (Aliverti et al., 2011; Fulco et al., 1994; Schmid et al., 2006). We replotted the data of Aliverti et al. (2011) to reflect perceived physical strain relative to peak exercise capacity at sea level and high altitude (rather than relative to peak power output at sea level)––perceived physical strain is equivalent at the same relative work rate at high altitude and sea level. However, it is not known how ascent to high altitude affects perceived mental strain relative to perceived physical strain.

Given that (a) the work rate performed is reduced at altitude for a given level of physical strain (Aliverti et al., 2011; Fulco et al., 1994; Schmid et al., 2006), and (b) the amount of central motor drive sent from the brain to the muscle—determining muscle activation—correlates with perceived mental strain (de Morree et al., 2012), we hypothesized that when exercising at the same relative exercise intensity perceived physical strain will remain unchanged whereas perceived mental strain will decrease on ascent from low to high altitude.

## 2 METHODS

### 2.1 Ethical approval

This study abided by the Canadian Government Tri‐Council policy on research ethics with human participants (TCPS2) and the Declaration of Helsinki, except for registration in a data base. Ethical approval was received in advance through Queen's University Health Sciences & Affiliated Teaching Hospitals Research Ethics Board (Protocol 6020426 PHE‐168‐17), Mount Royal University Human Research Ethics Board (Protocols 100012 and 100955) and was harmonized with the Nepal Health Research Council (Protocol 109‐2017). All participants were recruited via verbal communication and provided written and informed consent prior to voluntary participation in the study. All participants read and provided consent prior to their data being used for publication purposes.

### 2.2 Participants

Seventeen untrained young adults (8 males and 9 females) volunteered for the study. Four participants were excluded from analysis due to altitude‐ and travel‐related illnesses, while one participant was excluded because of missing data due to technology malfunction. All participants completed a physical activity readiness questionnaire (PAR‐Q) as well as filled out a health screening form prior to the beginning of the study.

### 2.3 Experimental procedure

**FIGURE 1:**
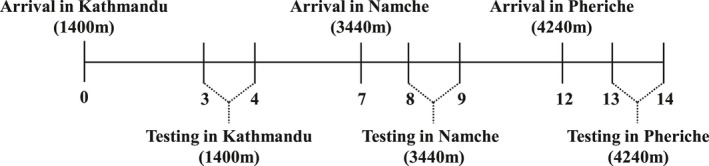
Timeline of high altitude exercise testing measured in days from arrival in Kathmandu. Numbering refers to the number of days since arrival in Kathmandu

The current study involved a subset of participants from a larger research expedition to Mount Everest base camp in Nepal. All low altitude testing was performed at 1400 m (Kathmandu), 3 days after arrival to allow for acclimation and recovery from travel (May 6, 2017). Four days later (May 10, 2017), after having hiked from 2840 m (Lukla) to 2840 m (Monjo) then to 3440 m (Namche) the second of the exercise tests was performed (see Figure 1 for summary of timeline). The final exercise test was performed 4 days later (May 14, 2017) after having hiked from 3440 m (Namche) to 3820 m (Deboche) and then to 4240 m (Pheriche). All participants hiked by foot and in accordance with the Wilderness Medical Society recommendations for low‐risk ascent the average altitude gained was <500 m per day (Luks et al., 2019), with rest days on day 3 (3440 m), day 5 (3820 m), and day 7 (4240 m), when the exercise protocols were performed. Daily resting physiological measurements were taken every morning between 06:00–08:00 throughout the trek to characterize responses to high altitude. The daily physiological measures consisted of resting heart rate (HR), blood pressure (10 Series Wireless Upper Arm Blood Pressure Monitor, Omron, Ontario, Canada), peripheral arterial oxygen saturation (SpO_2_; Masimo SET^®^ Rad‐5, Danderyd, Sweden), respiratory rate, and end‐tidal carbon dioxide (P_ET_CO_2_; EMMA, Masimo, Danderyd, Sweden).

### 2.4 Exercise protocol

The exercise protocol used in this study consisted of 20 min of moderate intensity walking exercise at a target HR reserve (HRR). Individual target heart rate ranges were calculated as 40%–60% of HRR using the Karvonen method (Karvonen & Vuorimaa, 1988). Resting HR measures were obtained every morning prior to exercise testing and were recorded via a wireless pulse oximeter. Participants were told their target HR zone and were given wrist‐worn HR monitors (HR; Polar FT1 or FT7, Polar Electro) to self‐monitor their HR during exercise. Exercise was completed at the same time of day within each participant at each elevation. Because of the crowded environment surrounding the guest house in Kathmandu, exercise at low altitude (Kathmandu, 1400 m) was completed on stairs in the hotel, whereas exercise at high altitude (Namche, 3440 m and Pheriche, 4240 m) was completed on walking paths outside near the guest house. HR, perceived physical strain and perceived mental strain were measured during exercise at minutes 5–7 (early) and 14–16 (late) of the 20 min walking test. Arterial oxygen saturation (Masimo SET^®^ Rad‐5) was recorded pre and post exercise. Core affect was only measured pre exercise to assess changes between altitudes (Venhorst et al., 2018a).

### 2.5 Perceptual scores

Measures of perceived physical and mental strain were obtained via qualitative self‐reporting scales that were developed by Swart et al. (2012). The physical rating of perceived exertion (P‐RPE) scale was a modified Borg 15‐point scale and was used to measure the perceived physical strain during exercise. Participants were to rate their perceived physical strain based on the feeling of how heavy and strenuous the exercise feels to them, combining all sensations and feelings of physical stress and fatigue. The task effort awareness (TEA) scale, a 15‐point scale developed by Swart et al. (2012), was used to measure perceived mental strain, defined as sensations related to the psychological/psychic effort required to continue the exercise bout at the chosen workload. Prior to testing, participants completed a familiarization questionnaire that explained the differences between perceived mental and physical strain, and a researcher debriefed participants following completion of the questionnaire to ensure comprehension. See Swart et al. (2012) supplemental files for exact instructions, explications, and questions asked for perceptual data collection.

Participants rated their P‐RPE and TEA scores twice during exercise, between 5–7 min (early exercise) and 14–16 min (late exercise). These scores were recorded at the end of an uphill portion of their walking exercise while HR was maintained within the predetermined HRR range. Once participants reached the top of this uphill portion, they were shown each scale sequentially and they were asked to point at or speak the number that represents their current sensation of perceived mental or physical strain. HR values were confirmed to lie within participants HRR range when TEA and P‐RPE were reported.

### 2.6 Core affect: Self‐Assessment Manikin

The Self‐Assessment Manikin is a 9‐point pictorial bipolar scale that was used to measure core affect—specifically happiness and arousal (Betella & Verschure, 2016; Bradley & Lang, 1994). Core affect refers to consciously affective feelings not directed to any specific object, physiological state or environment (Russell & Barrett, 1999). Prior to beginning exercise, each participant was shown the Self‐Assessment Manikin scale and was asked to point at the picture of Self‐Assessment Manikin that best represents their individual emotion of happiness and arousal. Self‐Assessment Manikin was measured to control for changes in core affect between altitudes that may alter an individual perception of mental or physical strain (Venhorst et al., 2018a). Core affect was not measured during exercise as to allow participants to focus on their perceptions of physical and mental strain.

### 2.7 Statistical analysis

Statistical tests were performed using SPSS version 24.0 software (SPSS Inc.). All data were found to be normally distributed (i.e., Kolmogorov‐Smirnov statistic >0.05). To test the main effects of altitude (1400, 3440, and 4240 m) and time on TEA, P‐RPE, exercising heart rate, and SpO_2_, two‐factor repeated measures analysis of variance with sex as between subject factor were completed to test for within‐subject differences. To test the main effect of altitude on happiness, arousal, average HR, resting HR, resting respiratory rate, resting partial pressure of end‐tidal CO_2_ and % heart rate reserve (%HRR), one‐way repeated measures ANOVAs with sex as between subject factor were performed. Partial η^2^ was calculated for all main effects and reported for all statistically different main effects. All pairwise comparisons were assessed post hoc via the Bonferroni method. Cohen's *d* was calculated for all significant post hoc pairwise comparisons. Statistical significance was assumed at *p* < 0.05. If assumptions of sphericity were violated, the Greenhouse–Geisser correction was used to estimate sphericity. Data are presented as means ± SD in the text and figures and individual participant data is presented in the same color in all figures.

## 3 RESULTS

The final sample presented in this study consisted of 12 young adults (8 females; age, 24 ± 4 years; height, 169 ± 11 cm; weight, 72 ± 17 kg).

### 3.1 Heart rate (HR)

#### 3.1.1 Resting HR

Resting HR increased with altitude [main effect of altitude on resting HR *F*(2, 20) = 8.89, *p* = 0.002, partial η^2^ = 0.470; Table 1] and this increase was not different between males and females [no altitude*sex interaction *F*(2, 20) = 0.44, *p* = 0.650].

**TABLE 1:** Change in physiological variables upon ascent from low to high altitude and accompanying pairwise comparisons

Variable	Altitude			Statistical Comparisons		
	1400m	3440m	4240m	1400m vs. 3440m	1400m vs. 4240m	3440m vs. 4240m
Resting HR (bpm)	83.33 ± 9.40	92.17 ± 12.66	81.75 ± 12.33	*p* = 0.022 *d* = 3.180	*p* = 1.00	*p* = 0.019 *d* = 0.583
Exercising HR (bpm)	151.37 ± 10.56	147.41 ± 16.37	144.28 ± 9.61	*p* = 0.951	*p* = 0.225	*p* = 1.00
HR Reserve (%)	55.25 ± 7.34	51.63 ± 6.70	50.17 ± 4.02	*p* = 1.00	*p* = 0.105	*p* = 0.272
SPO2 (%)	97.06 ± 0.16	90.66 ± 0.69	85.75 ± 1.11	*p* < 0.001 *d* = 8.071	*p* < 0.001 *d* = 14.262	*p* = 0.001 *d* = 9.803
P_ET_CO_2_ (Torr)	33.63 ± 0.96	27.63 ± 0.84	22.25 ± 0.71	*p* < 0.001 *d* = 7.103	*p* < 0.001 *d* = 13.479	*p* < 0.001 *d* = 5.965
TEA	3.44 ± 0.84	2.56 ± 0.84	2.05 ± 0.71	*p* = 0.268	*p* = 0.031 *d* = 1.787	*p* = 1.00
P‐RPE	10.87 ± 1.78	10.72 ± 1.29	11.41 ± 1.60	*p* = 1.00	*p* = 1.00	*p* = 0.281
Happiness	7.00 ± 1.41	6.75 ± 1.36	6.83 ± 0.83	*p* = 1.00	*p* = 1.00	*p* = 1.00
Arousal	5.67 ± 1.15	5.42 ± 1.08	6.25 ± 1.4	*p* = 1.00	*p* = 0.501	*p* = 0.225
AMS Scores	0.08 ± 0.29	1.00 ± 1.04	0.83 ± 0.94	*p* = 0.090	*p* = 0.153	*p* = 1.00

Abbreviations: AMS, altitude mountain sickness; *d*, Cohen's *d*; HR, heart rate; P_ET_CO_2_, partial pressure of end tidal carbon dioxide; P‐RPE, physical‐rating of perceived exertion; SpO_2_, arterial oxygen saturation; TEA, task effort awareness.

#### 3.1.2 Exercising HR

**FIGURE 2:**
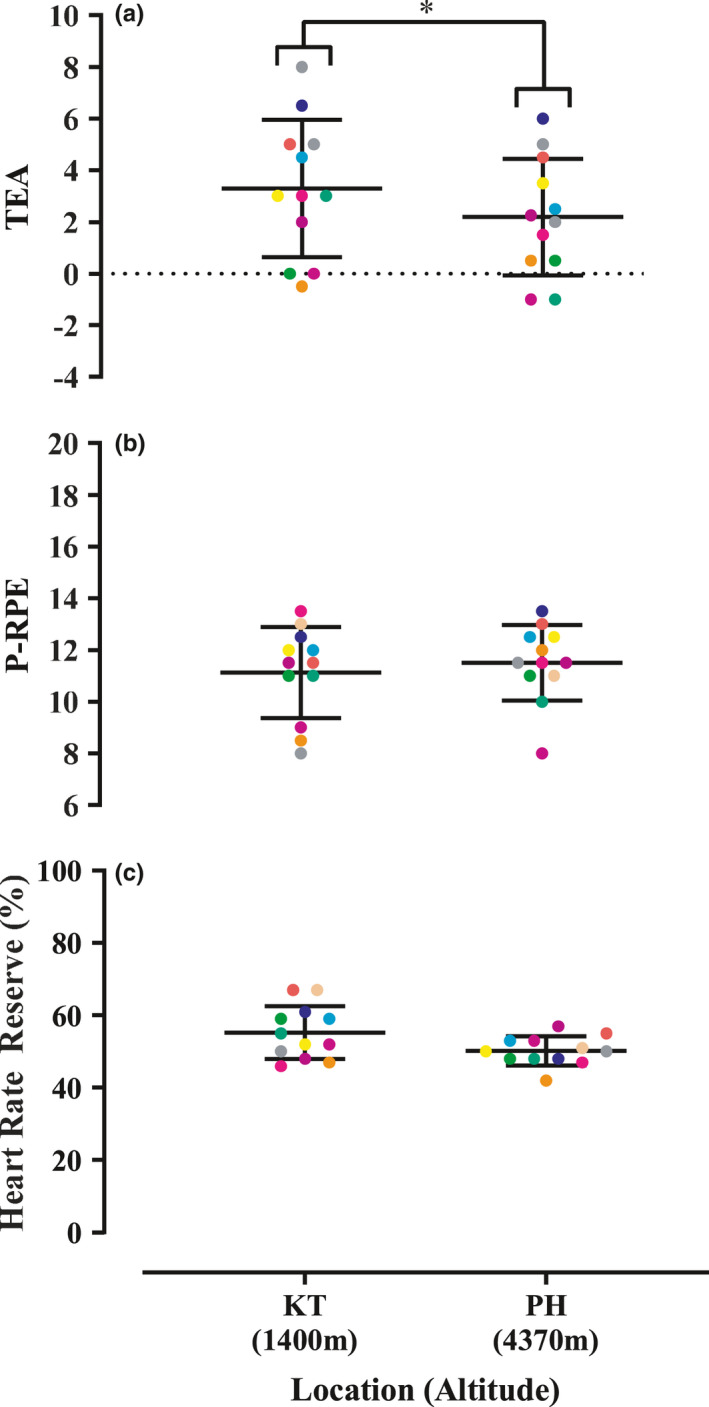
Perceived mental (Panel a) and physical (Panel b) strain measured with the task effort awareness (TEA) and physical‐rating of perceived exertion (P‐RPE) scales, respectively, during submaximal walking exercise at a targeted percent heart rate reserve (Panel c) in Kathmandu (KT, 1400 m) and Pheriche (PH, 4240 m). *Significantly different, *p* < 0.05

Exercising HR was not different between altitudes [no main effect of altitude *F*(2, 20) = 1.45, *p* = 0.257, see Table 1] or time [no main effect of time *F*(1, 10) = 0.98, *p* = 0.346] and there was no difference in exercising HR between males and females [no altitude*sex interaction *F*(2, 20) = 0.37, *p* = 0.691]. There were no other interactions [no time*sex *F*(1, 10) = 0.1.27, *p* = 0.286, no altitude*time *F*(2, 20) = 0.365, *p* = 0.698 or altitude*time*sex interaction *F*(2, 20) = 1.28, *p* = 0.300] on exercising HR. As intended, %HRR was not different between altitudes [no main effect of altitude *F*(2, 20) = 2.60, *p* = 0.099, see Figure 2] and there were no differences in %HRR between males and females as altitude increased [no altitude*sex interaction *F*(2, 20) = 1.00, *p* = 0.386, see Table 1 and Figure 2].

### 3.2 Core affect: Self‐Assessment Manikin

Altitude did not affect pre exercise arousal [*F*(2, 20) = 2.53, *p* = 0.105] or happiness [*F*(2, 20) = 0.19, *p* = 0.828] and there were no differences between males and females for arousal [no altitude*sex interaction *F*(2, 20) = 0.672, *p* = 0.522] or happiness [no altitude*sex interaction *F*(2, 20) = 0.12, *p* = 0.889, see Table 1].

### 3.3 Perceived physical strain as measured by physical‐rating of perceived exertion (P‐RPE)

P‐RPE was not different as altitude increased [no main effect altitude *F*(2, 20) = 1.16, *p* = 0.334, see Table 1 and Figure 2], nor did P‐RPE change with time [no main effect of time *F*(1, 10) = 0.842, *p* = 0.380] and there were no differences between males and females [no altitude*sex interaction *F*(2, 20) = 3.30, *p* = 0.275]. Furthermore, there was no time*sex interaction [*F*(1,10) = 0.30, *p* = 0.594], altitude*time [*F*(2, 20) = 0.14, *p* = 0.872], or altitude*time*sex interaction [*F*(2, 20) = 0.603, *p* = 0.524].

### 3.4 Perceived mental strain as measured by task effort awareness (TEA)

Whereas P‐RPE was maintained with increasing altitude, TEA decreased with increasing altitude [main effect of altitude *F*(2, 20) = 4.33, *p* = 0.027, partial η^2^ = 0.302; Table 1 and Figure 2] and this decrease was independent of sex [no altitude*sex interaction *F*(2, 20) = 2.97, *p* = 0.074, partial η^2^ = 0.229] and time [no main effect of time *F*(1, 1) = 0.50, *p* = 0.496] during exercise. There was no time*sex interaction [F(1, 10) = 0.42, *p* = 0.530] on TEA. There was no altitude*time interaction [F(2, 20) = 0.46, *p* = 0.637] nor was there an altitude*time*sex interaction [F(2, 20) = 3.04, *p* = 0.070] on TEA.

### 3.5 Arterial oxygen saturation (SpO_2_)

Resting SpO_2_ decreased as altitude increased [main effect of altitude *F*(2, 20) = 79.65, *p* < 0.001, see Table 1 for post hoc comparisons] and decreased pre‐ to post‐exercise [main effect of time *F*(1, 10) = 26.16, *p* < 0.001, Pre = 93.04 ± 0.44 vs. Post = 89.27 ± 0.84]. There was no altitude*sex [*F*(2, 20) = 2.34, *p* = 0.089] or time*sex interaction on SpO_2_ [*F*(1, 10) = 0.10, *p* = 0.762]. There was a significant altitude*time effect [*F*(2, 20) = 8.26, *p* = 0.002; Table 2] on SpO_2_ but there was no altitude*time*sex interaction [*F*(2, 20) = 1.48, *p* = 0.251].

**TABLE 2:** Post hoc analysis of significant interactions

Variable	Altitude	1400m	3440m	4240m
SpO_2_ (%)	Pre exercise	97.50 ± 0.52	93.67 ± 2.46	88.92 ± 3.31
	Post exercise	96.67 ± 0.98	88.42 ± 3.20	83.92 ± 5.78
	Statistical Comparisons	*p* = 0.034 *d *= 1.058	*p* < 0.001 *d* = 1.839	*p* = 0.005 *d* = 1.062
P_ET_CO_2_ (Torr)	Male	37.25 ± 2.36	29.00 ± 1.08	23.75 ± 1.25
	Female	30.00 ± 0.76	26.25 ± 1.05	20.75 ± 0.80
	Statistical Comparisons	*p* = 0.004 *d* = 4.125	*p* = 0.133	*p* = 0.062 *d* = 2.859

Abbreviations: *d*, Cohen's *d*; P_ET_CO_2_, partial pressure of end tidal carbon dioxide; SpO_2_, arterial oxygen saturation.

### 3.6 *Resting end*‐*tidal partial pressure of CO_2_ (P_ET_CO_2_)*

Resting P_ET_CO_2_ decreased as altitude increased [main effect of altitude *F*(2, 20) = 92,56, *p* < 0.001, partial η^2^ = 0.903, see Table 1 for post hoc comparisons] and these P_ET_CO_2_ measures were different between males and females [altitude * sex interaction *F*(2, 20) = 4.62, *p* = 0.022, partial η^2^ = 0.316, see Table 2].

### 3.7 Resting respiratory rate

Resting respiratory rate did not change with increasing altitude [no main effect of altitude *F*(2, 20) = 0.18, *p* = 0.833] nor was differ between males and females as altitude increased [no altitude*sex interaction *F*(2, 20) = 0.34, *p* = 0.715].

### 3.8 Altitude mountain sickness

There was a main effect of altitude on altitude mountain sickness [*F*(2,20) = 3.54, *p* = 0.048, partial η^2^ = 0.261] though post hoc analysis revealed no significant differences between altitudes (see Table 1).

## 4 DISCUSSION

The aim of this study was to test the hypothesis that when exercising at the same relative submaximal exercise intensity, perceived physical strain, and perceived mental strain will be dissociated with increasing altitude. Specifically, perceived physical strain will remain unchanged (i.e., same P‐RPE scores) whereas perceived mental strain will decrease (i.e., decreased TEA scores) as altitude increases during ascent from Kathmandu towards Mount Everest base camp in Nepal. The key findings from this study were: (1) perceived mental strain was highest at 1400 m (the lowest altitude) and decreased at 4240 m (the highest measured altitude, see Figure 2); (2) perceived physical strain (i.e., P‐RPE score) and %HRR during exercise were not different with increasing altitude (see Figure 2); and (3) changes in perceived mental strain were not because of changes in core affect (see Table 1). We interpret these key findings to support our hypothesis that during an ecologically valid walking exercise task, perceived physical strain remains unchanged and perceived mental strain decreases at the same relative exercise intensity as altitude increases during ascent from Kathmandu towards Mt Everest Base camp in Nepal.

### 4.1 Relative exercise intensity and perceived physical strain with increasing altitude

It is well established that maximal exercise capacity is decreased when exposed to high altitude and perceived physical strain scales with relative exercise intensity (Aliverti et al., 2011). Therefore, at the same perceived physical strain, absolute work rate is reduced whereas relative work rate is the same at sea level compared to high altitude.

Although previous work at high altitude has not had participants target a percent heart rate reserve (%HRR) as a means of setting a relative intensity, we have used the data of Schmid et al. (2006) to calculate the %HRR for participants exercising at a target power output during an incremental ramp cycling test at sea level and high altitude. These data were then plotted relative to their reported perceived physical strain (which these authors referred to as RPE leg). Although the slopes were observed to be significantly different, the perceived physical strain calculated at a %HRR of 50 (average of 40–60% targeted in our study) would be 10.94 and 11.53 at sea level and high altitude (delta = 0.59), respectively—reflecting a verbal anchor of “light” perceived exertion at the site of the leg. Interestingly, our participants’ rating of perceived physical strain fell within comparable levels at all altitudes when exercising at a similar %HRR (see Table 1). Our findings, compared to previous data, therefore support the use of %HRR as an effective approach for targeting a relative intensity.

By design, in our study, %HRR and perceived physical strain remained unchanged with increasing altitude (see Table 1 and Figure 2). Importantly, although we did not measure work rate, previous work strongly supports that absolute work rate would be reduced at the same relative intensity when exposed to high altitude (Aliverti et al., 2011), therefore, at the same perceived physical strain, work rate must have decreased as altitude increased in our study.

### 4.2 Dissociation of perceived mental from physical strain with increasing altitude

**FIGURE 3:**
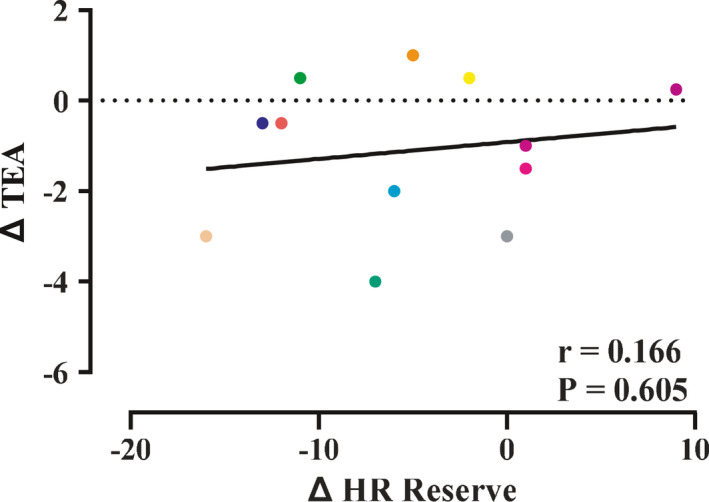
Change, from 1400 m to 4240 m, in percent heart rate (HR) reserve compared to perceived mental strain measured with the task effort awareness (TEA) scale

In contrast to perceived physical strain, perceived mental strain decreased with altitude (see Figure 2) and this change was not correlated with any change in %HRR observed during exercise (see Figure 3), nor was it due to a change in core affect (see Happiness and Arousal in Table 1). Given that perceived mental strain reflects central motor drive sent from the brain to the muscle determining muscle recruitment, if the absolute work rate is reduced—as would be expected at the same relative exercise intensity at altitude (Aliverti et al., 2011)––we would predict reduced central motor drive sent to the exercising muscle and therefore reduced perception of mental strain.

Although we cannot differentiate between an effect of acclimatization (adaptive and maladaptive) and hypoxia on perceived mental strain, previous work under hypoxic conditions strengthens our interpretation. Christian et al. (2014) had participants complete cycling exercise in hypoxia (F_I_O_2_ = 0.13, SpO_2_% = ~80) and normoxia (F_I_O_2_ = 0.21, SpO_2_% = ~97) targeting incremental levels of perceived mental strain [3–10 on a modified Borg CR10 scale, where perceived mental strain is referred by Christian et al. (2014) as perceived effort and defined as “the level of subjective awareness of effort expended during each exercise task”]. Importantly, the exercise task required participants to complete only 4 seconds of cycling exercise at each level of perceived mental strain, thereby eliminating any effects of hypoxia on skeletal muscle metabolic and contractile function that occur during sustained exercise (Allen et al., 2008; Allen & Trajanovska, 2012). Under their exercise conditions, Christian et al. (2014) found that at all levels of measured perceived mental strain, neither electromyography—an indirect measure of central motor drive (Amann & Dempsey, 2008; Amann et al., 2006)—nor mean power output were different between normoxic and hypoxic conditions. Consistent with such brief cycling bouts, there was also no difference in the amount of limb or overall peripheral discomfort in hypoxic versus normoxic cycling exercise at the same perceived mental strain, except for overall discomfort during the second of a repeated, maximal effort bout named “Max 2” (Christian et al., 2014). Thus, hypoxia does not appear to affect the relationship between central motor drive and perceived mental strain, which supports our interpretation that a reduction in perceived mental strain at high altitude may reflect a reduced central motor drive.

In summary, under ecological conditions of hiking from low to high altitude, which include acclimatization (adaptive and maladaptive) and hypoxia, this study has uncovered a dissociation of perceived physical and mental strain with increase in altitude. Specifically, mental strain decreases relative to physical strain at this submaximal relative exercise intensity.

### 4.3 Methodological considerations

First, due to participant dropout, our sample size is small and has an uneven number of male and female participants. We acknowledge that there is a lack of statistical power to detect differences between sexes, therefore, we have provided sex comparisons as exploratory. Considering there are sex differences in performance fatigability (Hunter, 2014, 2018), further examination of perceived fatigability between sexes at altitude is needed. Next, due to the small sample size, we did not detect a difference in %HRR between altitudes. A sample size calculation from our data reveals that we would have needed 42 participants to detect a change in %HRR between altitudes—this provides greater confidence that there is no physiologically relevant difference in %HRR between altitudes.

Second, we used a relatively wide %HRR range for targeting our relative exercise intensity––this could have resulted in differences in the mean %HRR at different altitudes. Consistent with %HRR being similar across altitudes, perceived physical strain did not change as altitude increased––yet perceived mental strain did change. Combined, these data support our %HRR range as being a tight enough constraint to reveal dissociation of perceived mental and physical strain. Furthermore, if %HRR differences were responsible for our observed findings, we should see a correlation between the changes in %HRR and perceived mental strain across altitudes––there was no correlation (see Figure 3).

Third, due to the crowded environment surrounding the guest house in Kathmandu, exercise at low altitude (Kathmandu, 1400 m) was completed on stairs in the hotel, whereas exercise at high altitude (Namche, 3440m and Pheriche, 4240m) was completed on walking paths outside near the guest house. During exercise, participants were constantly self‐monitor their HR and adjusting their pace to maintain their HR within the predetermined HRR range. This constant monitoring of HR during exercise reduces the likelihood of differences in exercise environment contributing to the changes in perceived mental strain.

## 5 CONCLUSION

Recent work has highlighted the importance of assessing not only performance but also perceived fatigability. Until now, no work had examined the domain of perceived mental strain upon ascent from low to high altitude. Accordingly, this study demonstrates for the first time that as altitude increases, perceived mental strain dissociates from perceived physical strain during relative intensity submaximal exercise. In fact, perceived mental strain decreases at the same perceived physical strain as altitude increases on ascent from Kathmandu to mount Everest base camp in Nepal. A follow‐up on this work is therefore warranted to identify the mechanism(s) responsible for the observed dissociation between perceived mental and physical strain upon ascent from low to high altitude.

## CONFLICT OF INTEREST

No conflict of interest, financial, or otherwise, are declared by the authors.

## AUTHOR CONTRIBUTIONS

P.J.D., J.J.W., and M.E.T. conceived and designed the research; P.J.D. and J.J.W. performed the experiments; P.J.D. analyzed the data; P.J.D., J.J.W, J.S., T.A.D., and M.E.T. interpreted the results of experiments; P.J.D. prepared the figures and tables; P.J.D. drafted the manuscript; P.J.D., J.J.W, J.S., T.A.D., and M.E.T. edited and revised the manuscript; P.J.D., J.J.W, J.S., T.A.D., and M.E.T. approved the final version of manuscript.
